# Non-exercise, home-based interventions for persons living with dementia: A commentary on a systematic review

**DOI:** 10.12968/bjnn.2024.0030

**Published:** 2025-12-02

**Authors:** P Somner, J Harrison, J Hill

**Affiliations:** Lancashire and South Cumbria NHS Foundation Trust; University of Lancashire

**Keywords:** Dementia, carers, home-based activities, non-exercise

## Abstract

Dementia is a neurological condition that is estimated to effect over 55 million people globally. For people living with dementia, maintaining meaningful activity adds to quality of life, maintaining purpose and routine. The evidence for activity interventions that were home based, and did not focus on physical exercise, was unclear for persons living with dementia. A systematic review by Tan et al. 2022 consolidated the evidence of effectiveness for such interventions, for both the person living with dementia and their carer. This commentary critically appraises the review and discusses the findings for health and social care professionals in dementia care.

## Introduction

Dementia is a progressive, multi-faceted neurological condition which destroys nerve cells in the brain, thus effecting memory, thinking, and abilities to undertake tasks in everyday life (James & Jackman, 2017; WHO, 2023). Currently, it is estimated that the number of people living with a diagnosis of dementia has exceeded over 55 million worldwide (WHO, 2023). Considering the high likelihood of unreported cases, this figure is likely to be even higher (Parker et al, 2022). In the UK, there are an estimated 900,000 older people living with the condition (Wittenberg et al, 2019).

For a person living with dementia (PLWD), maintaining meaningful activity adds to quality of life, provides purpose and routine, acknowledges skills and life experiences, maintains independence, and gives the opportunity for more social time (Harrison-Denning, 2021). Based on the perspectives of PLWD, engagement in personally meaningful activities enables connection with self, with others, and with the environment, underscoring the importance of person-centred activities (Han et al. 2016). In the UK, the Prime Minister’s challenge on dementia 2020 requested greater provision of personalised care, to help people with dementia both at home and in care remain active and engaged, with regular opportunities for social interaction and activities focused on the individual (DoH, 2015).

A systematic review by Tan et al. (2022) aimed to understand the effectiveness of home-based, non-exercise interventions for PLWD and their carers. The aim of this commentary is to appraise the quality of the systematic review, and to subsequently explore the implications of the findings for both practice and future research.

## Methods of the review by Tan et al. (2022)

A comprehensive, electronic, and multi-database search was undertaken from inception to June 2021, in addition to hand searching of reference lists. Screening of titles, abstracts and full-text articles was undertaken by three reviewers. Studies were included if participants lived at home with a medical diagnosis of dementia, and utilised individual interventions, including a component other than physical exercise, and within the home setting. Included studies were required to be randomised controlled trials (RCTs) or quasi-randomised trials (QR), published in English and within a peer-reviewed journal. Quality assessment was undertaken by two reviewers using the JBI checklist for RCTs and quasi-experimental studies. Data extraction was undertaken by three reviewers independently, and verified by one further reviewer. Data were synthesised based on the direction of effects reported, and grouped into categories of: behavioural disturbance, cognition, mood, functional status and effects on caregivers (quality of life, mood, carer burden).

## Results of the review by Tan et al. 2022

From a comprehensive synthesis of 3,882 studies, 97 full text studies were assessed for eligibility, of which 18 studies were selected (14 RCTs and 4 QR studies). The studies represented interventions that included cognitive rehabilitation (n=3), tailored activity programmes (n=3), cognitive stimulation (n=2), occupational therapy (n=2), reminiscence (n=1), music therapy (n=1) reality orientation (n=1), biobehavioural intervention (n=1), and multi-component intervention (n=4). A total of 1520 people with a diagnosis of dementia and 1420 carers were included in the 18 studies. An overall assessment of study quality was not provided, however it was reported that no RCTs reported either blinding of participants or those who delivered the treatment. The reliability of the data collection was also reported to be unclear for both RCTs and quasi-experimental studies.

### Behavioural disturbance

There was mixed evidence for the impact of interventions on behavioural disturbance. Three RCTs using tailored activity programmes, and one QR of music therapy, reported a significant reduction compared to the control groups relative to baseline. Specific to agitation, only one study using a tailored activity programme found a significant decrease, and a further six RCTs reported no significant effect (reality orientation, cognitive rehabilitation, individualized cognitive stimulation therapy, multi-component). Of those interventions that were effective, the use of tailored activities such as the interests, capabilities, and dyadic needs of the PLWD were a common feature, as was psychoeducation and skills training for carers (communication and task simplification). A trend was also noted for interventions specifically designed to manage or target symptoms of behaviour indicating significant improvements, as opposed to those that did not.

### Cognition

Eight studies reported the effects of interventions on cognition outcomes. An RCT and a QR study investigating psychoeducation, memory rehabilitation, and reality orientation reported significant benefits to cognition. Mixed results were reported by two interventions of cognitive rehabilitation and multi-component intervention. Four RCTs reported no evidence of difference when exploring the effects of individualized cognitive stimulation therapy or cognitive rehabilitation interventions. Where improvements to cognition were indicated, the common features of interventions were reinforced strategies in between sessions, carers delivering interventions, and studies with longer durations (>8 weeks).

### Mood

Six RCTs investigating interventions of tailored activities, individualized cognitive stimulation therapy, cognitive rehabilitation, and multi-component interventions reported on mood outcomes, with no significant effect identified relative to comparison groups. One RCT noted that depression increased when intervention tasks were too challenging. No specific outcomes for positive emotions were measured.

### Functional Status

Mixed results from 11 RCTs and two QR studies were reported for functional status. Improvements in functional abilities were noted in five RCTs and one QR study, where the intervention was either occupational therapy, biobehavioural intervention, cognitive rehabilitation or a tailored activity programme. All six of these interventions included a pre-intervention assessment for ability, used individualised activities aligned to interests, and taught specific skills to PLWD and carers to perform functional activities. One RCT study of cognitive rehabilitation reported mixed results, and the remaining five RCTs and one QR study found no significant improvements (cognitive rehabilitation, individualized cognitive stimulation therapy, reality orientation, music therapy and multi-component).

### Caregiver’s Quality of Life

Five RCTs and two QR studies considered the effect of interventions on caregiver’s quality of life. A higher quality of life was reported for caregivers in a single RCT and QR study, relative to the control groups, and after receiving a biobehavioural intervention and a multi-faceted intervention respectively. Both interventions involved the caregivers. Mixed results were reported in three RCTs of cognitive rehabilitation and individualized cognitive stimulation therapy, with improvements in some domains such as social relationships. A single RCT and QR study indicated no significant improvements (reminiscence therapy and reality orientation). Overall, the activities that were the most effective actively involved caregivers, providing they were not over intensive or too demanding.

### Caregiver’s Mood

Overall, there were no significant improvements reported for carer mood when measured in six RCTs and one QR study of tailored activity programmes, individualized cognitive stimulation therapy, cognitive rehabilitation, and reality orientation.

### Caregiver’s Burden

Eight studies explored the impact on carer burden. No significant effects on carer burden were found in four RCTs and one QR of tailored activity programmes, cognitive rehabilitation, music therapy, and reality orientation. Mixed results were reported by one RCT of a tailored activity programme. A single RCT and QR study reported that individualised occupational therapy showed a decrease in carer burden compared to control groups. Both studies included caregiver education such as teaching care and coping strategies, and observed improvements in the functional ability of PLWD.

## Commentary

The critique of a systematic review, summarising both qualities and limitations, helps to determine if a systematic review is sufficiently robust to influence clinical practice, and to improve standards of reliable evidence (Frewen et al. 2023). The AMSTAR 2 tool, a critical appraisal tool for systematic reviews (Shea et al, 2017), was utilised to critically appraise the methods used within Tan et al. 2022. Eleven of the 16 AMSTAR criteria were satisfied, partially satisfied or non-applicable (i.e. no meta-analysis was conducted). The five criteria which were unclear or unsatisfied were: no clear statement of the review methods provided in a pre-study protocol, no details of excluded studies (reasons for exclusion provided but no list given), no justification for including English language only studies (described as a limitation but no reason given), no evidence provided relating to sources of funding for included studies, and no interpretation of the quality of the studies, and what this means for the results (e.g. no summary provided of whether studies were mostly of good or poor quality). Hence, although this review provides a comprehensive overview of the research for non-exercise, home-based interventions for PLWD, due to the limitations listed some caution should be applied when interpreting the results for practice.

The review authors concluded that although the evidence for the effectiveness of home-based, non-exercise interventions for PLWD was mixed, important intervention features were highlighted. The most prominent of these were: personalised activities aligned to the interests and ability of PLWD, actively involving caregivers in activities, and psychoeducation or skills training for carers in communication, task simplification and performing functional activities.

### Personalised activities aligned to the interests and ability of PLWD

Personalised or tailored activities were a notable feature of successful interventions. Likewise, a review by Mohler et al. 2023 found that personally tailored interventions may slightly reduce agitation in PLWD, while Travers et al. 2016 identified that tailored and meaningful activities for PLWD in residential care appeared effective for a range of behavioural and psychological symptoms. These activities included a preferred music intervention for agitation, depression, and anxiety, and reminiscence therapy for mood and cognition (Travers et al, 2016). Current guidelines defined by the National Institute for Health and Care Excellence (NICE, 2018; NICE, 2019) align with these findings, and state that PLWD should be supported to choose from a range of personalised activities to support engagement, pleasure and interest. The Global action plan on the public health response to dementia 2017-2025 also proposes a pathway for person-centred care and meaningful activities (WHO, 2017). However, 37% of PLWD from higher income countries and 45% from lower income countries are not offered post-diagnosis support beyond initial information (ADI, 2022). Dementia service providers and practitioners should aim to discuss with PLWD the tailored activities they wish to access, including their preferences, needs, life experiences, circumstances, and interests (NICE, 2019).

### Active involvement of caregivers in activities

Actively involving carers in activities was a notable feature of interventions where the functional status of PLWD, and carer quality of life was improved. For PLWD, connection with others provides motivation to engage in activities and to feel connected Han et al. (2016). By engaging PLWD in everyday and enjoyable activities at home, carers and families also help to maintain the person’s agency and personhood, creating stability and continuity (Chung et al, 2017). Social engagement and connection with others are an element of living well with dementia, and can be supported professionally by an assessment of need and wellbeing (Quinn et al, 2021). Current guidance suggests that family members or carers should be offered advice on planning enjoyable and meaningful activities to do with the person they care for, in addition to involvement in decision making, and developing care-plans (NICE, 2018). Including carers in care and treatment will offer better outcomes for PLWD and enable staff and services to have a full picture of the person’s needs (Carers Trust, 2016).

### Psychoeducation or skills training for carers

Psychoeducation or skills training for carers in communication, task simplification or performing functional activities, was a prominent feature of effective interventions for behavioural disturbance, functional status and reducing caregiver burden. Providing carers with dementia education and training, carer stress, and coping strategies (including emotional and psychological support), has previously been shown to reduce carer depression, enhance carer quality of life, and reduce costs through a decreased use of services (Livingston et al, 2014). Dementia caregivers who have attended a psycho-educational program also experience a more positive outlook towards dementia, having improved knowledge, confidence in care, and lowered anxiety (Teichmann et al. 2022). Additionally, it has been indicated that if care partners remain well and receive adequate training and support, PLWD in the community will continue to engage in meaningful activities (Allison et al, 2022). Guidelines state that the carers of PLWD should be offered psychoeducation and skills training, including dementia education and personalised strategies, carer skills, advice on planning enjoyable and meaningful activities, future planning and their own wellbeing (NICE, 2018). For the requirements of future practice, the Dementia Training Standards Framework (SfH and HEE, 2018), would benefit from an update, to ensure that informal carers and families receive consistent standards of training and support to meet their needs. Additional resources for carer education may be available through carer support groups or courses, helping informal carers be better informed, prepared and supported (Atkinson et al, 2022). Local dementia hubs may also provide awareness sessions, group events and a place of support for both carers and PLWD (Trinity Hospice, 2017).

## Conclusion

Although findings from the review by Tan et al. 2022 were mixed, there were notable features of effective home-based, non-exercise interventions for PLWD: personalised or tailored activities, active involvement of caregivers and caregiver education. Further research is required to verify these moderating factors.

### Reflective questions

- What factors influence the provision of tailored or personalised activities for PLWD and the active involvement of caregivers?
- What resources are available for carer education, training and support in dementia care?

## Key Points

- The evidence for the effectiveness of home-based, non-exercise interventions on behavioural disturbance, cognition and functional status is mixed for people living with dementia, with no effect on mood.
- For carers, the evidence is also mixed for quality of life and caregiver burden, with no effect reported for carer mood.
- Personalised tailored activities, active involvement of the caregiver, provision of caregiver education or training, were the most notable features of effective interventions.
